# Supplementary material for: Host–Guest Interactions of Zirconium-Based Metal–Organic Framework with Ionic Liquid
Source: Molecules. 2023 Mar 21;28(6):2833. doi: 10.3390/molecules28062833 (PMC10055841; doi:10.3390/molecules28062833)
Supplement: Supplementary file 1 [file molecules-28-02833-s001.zip › molecules-2262724-supplementary.pdf]

Supplementary Information

Host-Guest Interactions of Zirconium-based Metal-Organic Framework with Ionic Liquid

Mohd. Faridzuan Majid [1,2], Hayyiratul Fatimah Mohd Zaid [2,3,*], Muhammad Fadhlullah Abd Shukur [1,2], Azizan Ahmad [4,5] and Khairulazhar Jumbri [1,6]

1 Department of Fundamental and Applied Sciences, Universiti Teknologi PETRONAS, 32610 Seri Iskandar, Perak Darul Ridzuan, Malaysia; mohd._17006281@utp.edu.my (M.F.M.); mfadhlullah.ashukur@utp.edu.my (M.F.A.S.); khairulazhar.jumbri@utp.edu.my (K.J.)
2 Centre of Innovative Nanostructures & Nanodevices (COINN), Universiti Teknologi PETRONAS, 32610 Seri Iskandar, Perak Darul Ridzuan, Malaysia
3 Chemical Engineering Department, Universiti Teknologi PETRONAS, 32610 Seri Iskandar, Perak, Malaysia
4 Department of Chemical Sciences, Universiti Kebangsaan Malaysia, 43600 Bangi, Selangor, Malaysia; mfadhlullah.ashukur@utp.edu.my
5 Department of Physics, Faculty of Science and Technology, Airlangga University (Campus C), Mulyorejo Road, Surabaya 60115, Indonesia
6 Centre for Research in Ionic Liquids (CORIL), Universiti Teknologi PETRONAS, 32610 Seri Iskandar, Perak Darul Ridzuan, Malaysia
* Correspondence: hayyiratul.mzaid@utp.edu.my; Tel.: +605-3687618

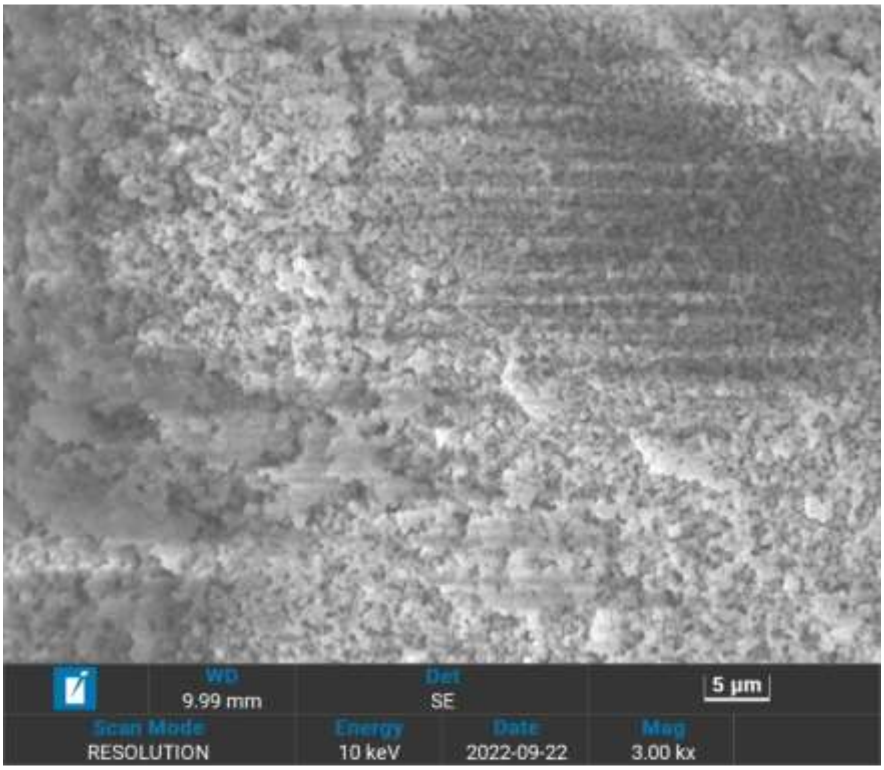

Figure S1. The surface of pristine UiO-66 at 5 μm.

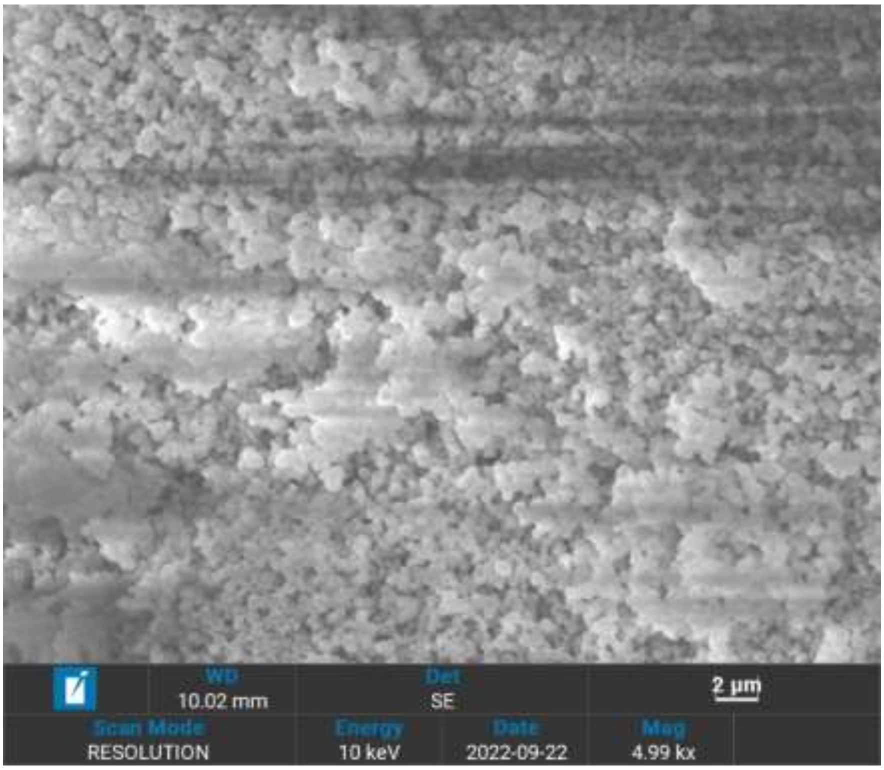

Figure S2. The surface of pristine UiO-66 at 2 μm.

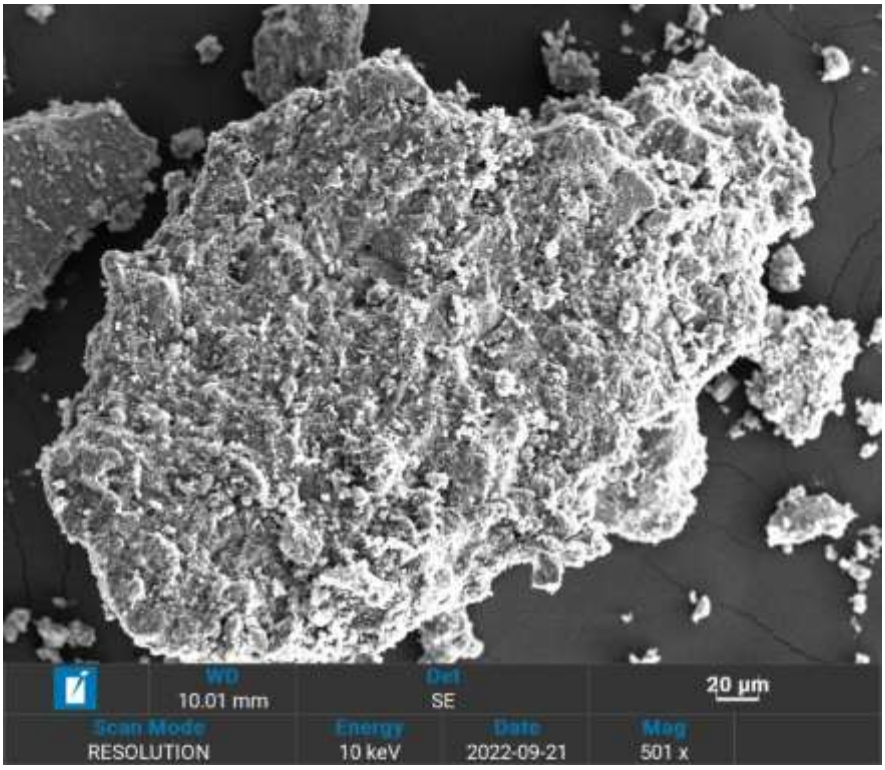

Figure S3. The surface UiO-66@IL at 20 μm.

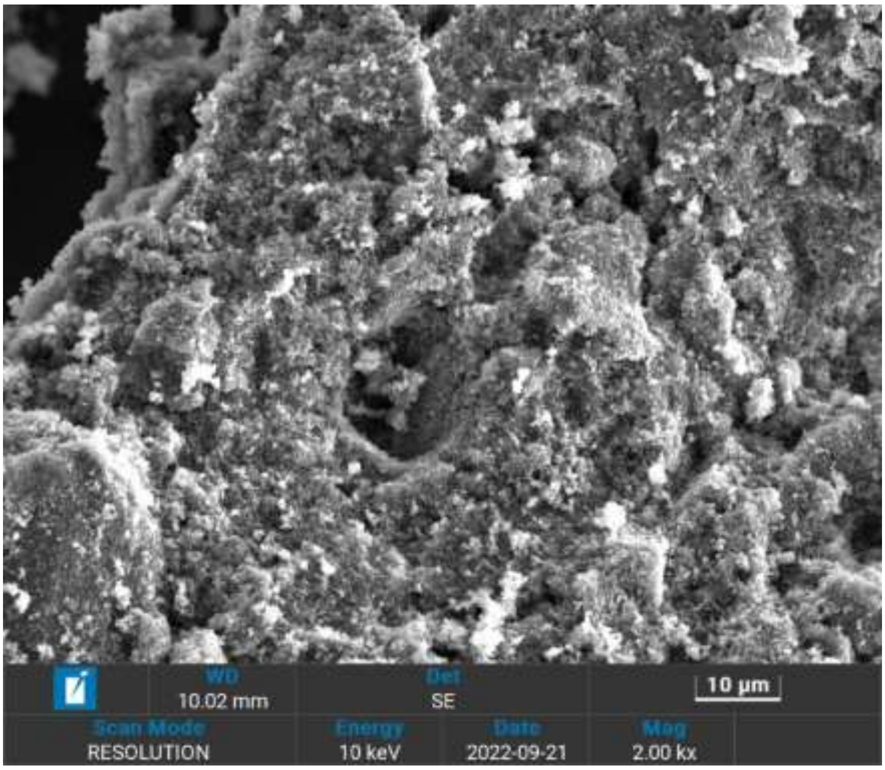

Figure S4. The surface of UiO-66@IL at 10 μm.
